# Regulation of alternative VEGF-A mRNA splicing is a therapeutic target for analgesia^☆^

**DOI:** 10.1016/j.nbd.2014.08.012

**Published:** 2014-11

**Authors:** R.P. Hulse, N. Beazley-Long, J. Hua, H. Kennedy, J. Prager, H. Bevan, Y. Qiu, E.S. Fernandes, M.V. Gammons, K. Ballmer-Hofer, A.C. Gittenberger de Groot, A.J. Churchill, S.J. Harper, S.D. Brain, D.O. Bates, L.F. Donaldson

**Affiliations:** a Physiology and Pharmacology, University of Bristol, Bristol BS8 1TD, UK; b King's College London, London SE1 9NH, UK; c Paul Scherrer Institut, 5232 Villigen, Switzerland; d Anatomy and Embryology, Leiden University Medical Centre, 2300 RC Leiden, The Netherlands; e Clinical Sciences, University of Bristol, Bristol BS1 2LX, UK; f Cancer Biology, Division of Cancer and Stem Cells, School of Medicine, University of Nottingham, Queen's Medical Centre, Nottingham NG2 7UH, UK; g School of Life Sciences, The Medical School, University of Nottingham, Queen's Medical Centre, Nottingham NG2 7UH, UK

**Keywords:** VEGF-A, vascular endothelial growth factor-A, SRPK1, serine arginine protein kinase 1, SRSF1, serine arginine splice factor 1, VEGFR2, vascular endothelial growth factor receptor 2, IB4, isolectin B4, TRPV1, transient receptor potential vanilloid 1, CV, conduction velocity, PSNI, partial saphenous nerve ligation injury, DRG, dorsal root ganglia, Vascular endothelial growth factor A, Alternative mRNA splicing, Neuropathy, Nociceptors

## Abstract

Vascular endothelial growth factor-A (VEGF-A) is best known as a key regulator of the formation of new blood vessels. Neutralization of VEGF-A with anti-VEGF therapy e.g. bevacizumab, can be painful, and this is hypothesized to result from a loss of VEGF-A-mediated neuroprotection. The multiple *vegf-a* gene products consist of two alternatively spliced families, typified by VEGF-A_165_a and VEGF-A_165_b (both contain 165 amino acids), both of which are neuroprotective. Under pathological conditions, such as in inflammation and cancer, the pro-angiogenic VEGF-A_165_a is upregulated and predominates over the VEGF-A_165_b isoform.

We show here that in rats and mice VEGF-A_165_a and VEGF-A_165_b have opposing effects on pain, and that blocking the proximal splicing event – leading to the preferential expression of VEGF-A_165_b over VEGF_165_a – prevents pain in vivo. VEGF-A_165_a sensitizes peripheral nociceptive neurons through actions on VEGFR2 and a TRPV1-dependent mechanism, thus enhancing nociceptive signaling. VEGF-A_165_b blocks the effect of VEGF-A_165_a.

After nerve injury, the endogenous balance of VEGF-A isoforms switches to greater expression of VEGF-A_xxx_a compared to VEGF-A_xxx_b, through an SRPK1-dependent pre-mRNA splicing mechanism. Pharmacological inhibition of SRPK1 after traumatic nerve injury selectively reduced VEGF-A_xxx_a expression and reversed associated neuropathic pain. Exogenous VEGF-A_165_b also ameliorated neuropathic pain.

We conclude that the relative levels of alternatively spliced VEGF-A isoforms are critical for pain modulation under both normal conditions and in sensory neuropathy. Altering VEGF-A_xxx_a/VEGF-A_xxx_b balance by targeting alternative RNA splicing may be a new analgesic strategy.

## Highlights

• The different *vegf-a* splice variants, VEGF-A_165_a and VEGF-A_165_b have pro- and anti-nociceptive actions respectively.
• Pro-nociceptive actions of VEGF-A_165_a are dependent on TRPV1.
• Alternative pre-mRNA splicing underpins peripheral sensitization by VEGF-A isoforms in normal and neuropathic animals.

## Graphical abstract

**Figure:**
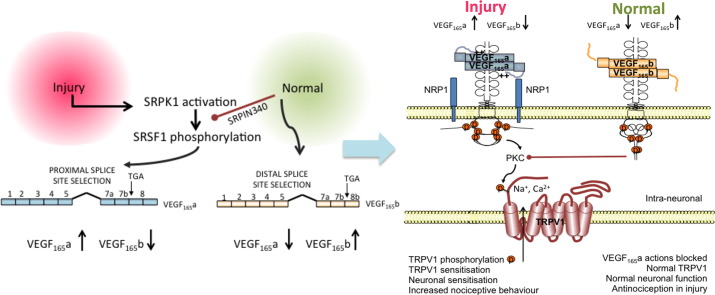

## Introduction

Neutralization of VEGF-A with anti-VEGF-A therapies, such as bevacizumab or VEGF-A receptor inhibitors (e.g., vandetanib) can result in pain, when given alone (Burger et al., 2007, Cohen and Hochster, 2007) or in combination with chemotherapies (Cohen et al., 2007, Garcia et al., 2008, Langenberg et al., 2011, Miller et al., 2007). The clinical findings that VEGF-A contributes to pain are supported by observations that inhibition of VEGF receptor 2 (VEGFR2) exacerbates peripheral neuronal damage, which is often associated with pain (Beazley-Long et al., 2013, Verheyen et al., 2012), and enhances pain behaviors in normal, nerve-injured and diabetic animals (Hulse et al., 2010a, Verheyen et al., 2012).

**Fig. 1:**
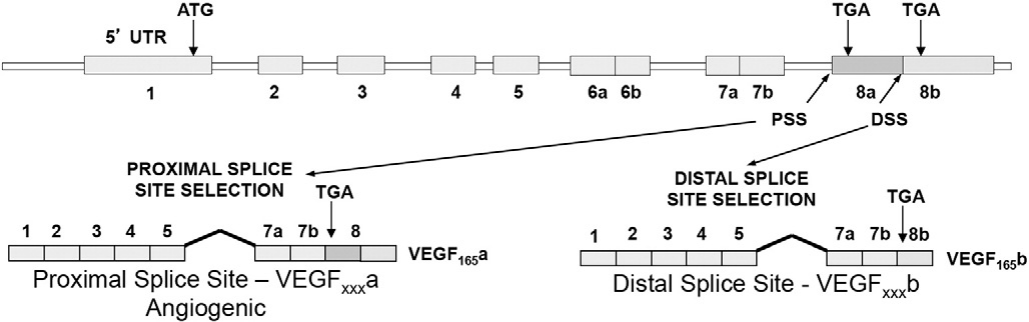
VEGF-A gene splice variant isoforms. VEGF-A pre-mRNA is alternatively spliced to form two families of mRNAs: VEGF-A_xxx_a and VEGF-A_xxx_b. The archetypal forms VEGF-A_165_a and VEGF-A_165_b are shown for illustration. VEGF-A_xxx_a proteins are translated from mRNAs that use the proximal splice site (PSS) and include all of exon 8, VEGF-A_xxx_b proteins from mRNAs that use the distal splice site (DSS) and contain only the b part of exon 8. The neuropilin-1 (NP-1) co-receptor binding site is located at the distal end of exon 7 and proximal exon 8a.

The *vegf-a* gene encodes two families of isoforms typified by VEGF-A_165_ a and VEGF-A_165_b (Harper and Bates, 2008). Both families have sister isoforms of the same length so they are referred collectively as VEGF-A_xxx_a and VEGF-A_xxx_b where xxx represents the number of amino acids. The isoform families differ only in their six C terminal amino acids (Harper and Bates, 2008), and they are both capable of binding to VEGFR2 with similar affinities, but the functional results of receptor activation are multivariate (Table 1) (Ballmer-Hofer et al., 2011). Control of relative isoform expression occurs by alternative pre-mRNA splicing of either proximal or distal splice sites in exon 8 (Fig. 1).

**Table 1:** Overview of the C-terminal sequences, binding domains and interactions with VEGFR2 of the different VEGF-A splice variant isoforms.

	C terminal sequence	Binding domains present	Consequences of receptor binding
VEGF-A_165_a	CDKPRR	VEGFR1, VEGFR2, NP-1	Full agonist. Binds and stabilizes VEGFR + NP-1 interaction. Complete phosphorylation at Y1175. PIP2 hydrolysis, PKC activation.
VEGF-A_165_b	SLTRKD	VEGFR1, VEGFR2	Partial VEGFR2 agonist/competitive inhibitor of VEGF-A_165_a binding. Very weak NP-1 interaction. Weak/incomplete phosphorylation at Y1175 No PIP2 hydrolysis, or PKC activation. Receptor internalization and degradation. (Ballmer-Hofer et al., 2011, Kisko et al., 2011)
VEGF-A_159_	–	VEGFR1, VEGFR2	Binds VEGFR, no activation. Very weak NP-1 interaction.
VEGF-A_121_a	CDKPRR	VEGFR1, VEGFR2	Binds VEGFR. Very weak NP-1 interaction. Complete phosphorylation at Y1175

VEGF-A_xxx_a is the principal target of anti-VEGF and VEGFR therapies as these isoforms are upregulated and predominate in many pathologies. However, VEGF-A_xxx_b forms a significant proportion of total (pan-)VEGF-A protein in many normal tissues (Harper and Bates, 2008) so the therapeutic effects of VEGF-A sequestration with many current antibody therapies, or VEGFR2 inhibition are a net result of simultaneous blockade of the actions of *both* families. The impact of the neutralization of the VEGF-A_xxx_b family on treatment outcomes has only recently been exemplified, in terms of its ability to predict colorectal cancer patients that do not respond to bevacizumab (Bates et al., 2012).

rhVEGF-A_165_a exacerbated spinal cord contusion-associated pain and damage (Benton and Whittemore, 2003, Herrera et al., 2009, Nesic et al., 2010, Sundberg et al., 2011), and referred mechanical abdominal pain (Malykhina et al., 2012), but local VEGF-A delivery (presumed VEGF-A_xxx_a) partially reversed diabetic neuropathic mechanical hyperalgesia (Verheyen et al., 2013). Neutralization of all endogenous VEGF-A isoforms or VEGF receptor 2 inhibition increased pain sensitivity in chemotherapy-induced neuropathy (Verheyen et al., 2012), but conversely reversed neuropathic (Lin et al., 2010), and acute inflammatory hyperalgesia (Grosios et al., 2004).

These conflicting observations might be explained by different actions of the distinct isoforms, which have not been studied independently, and their differing actions on VEGFR2 (Ballmer-Hofer et al., 2011). We therefore tested the hypothesis that the alternatively spliced VEGF-A isoform families have different effects on pain. We investigated: a) the effects of specific VEGF-A isoforms on pain/nociception; b) the neuronal mechanisms through which effects on pain might occur; c) whether using control of alternative RNA splicing of VEGF-A could modulate nociception/pain, and d) whether either VEGF-A proteins or alternative splicing control may be potential novel analgesic targets.

## Materials and methods

All procedures using animals were performed in accordance with the United Kingdom Animals (Scientific Procedures) Act 1986 and with University of Bristol and King's College London Ethical Review Groups approval. Human embryonic and adult tissues were obtained under ethical approval by University of Leiden and adult human DRG under ethical approval by Southmead Hospital Local Research Ethics Committee.

### Antibody and pharmacological inhibitors

The following pharmacological interventions were used: pan-VEGF-A neutralization with mouse anti-VEGF-A antibody (Liang et al., 2006), specific VEGF-A_165_b neutralization using systemic treatment with anti-VEGF-A_165_b antibody (clone 56/1, (Woolard et al., 2004)) systemic and local VEGF receptor inhibition with selective (PTK787; (Wood et al., 2000)) and/or specific (ZM323881; (Whittles et al., 2002)). VEGFR2 tyrosine kinase inhibitors; systemic or local administration of VEGF-A_165_a and/or VEGF-A_165_b; systemic antagonism of TRPV1 with SB366791 (Varga et al., 2005); inhibition of serine-rich protein kinases with SRPIN340 (Fukuhara et al., 2006), and appropriate vehicles.

### Measurement of mechanical and thermal nociceptive behaviors

A total of 64 adult male mice (C57Bl6, 25–30 g), 6 TRPV1 congenic knockouts and 6 wild-type strain-matched controls and 24 adult male Wistar rats were used to assess nociceptive behavior. TRPV1 homozygous knockout mice breeding pairs were generated and bred as described at King's College London, (Caterina et al., 2000, Fernandes et al., 2011, Fernandes et al., 2013) where breeding colonies were regularly backcrossed according to Jackson Laboratory guidelines to avoid sub-strain selection (Lambert, n.d.).

All animals were habituated to testing environments and handling prior to testing, and were allowed to habituate to the environment for at least 15 min at each test session. Nociceptive testing, as previously described (Hulse et al., 2008), consisted of measurement of mechanical allodynia by determination of von Frey hair mechanical withdrawal threshold and thermal hyperalgesia using the Hargreaves test (Hargreaves et al., 1988). Behavioral testing groups were randomized, and all operators were blinded to the drug and surgical treatment (nerve injury/sham) in each animal in all experiments.

### Von Frey hair mechanical thresholds — mechanical allodynia

Animals were habituated to chambers with mesh floors. The plantar surface of each foot was stimulated with von Frey hairs (Linton, UK) of increasing gram force breaking points, over a range of 0.07–2 g (mice), or 1–100 g (rats) (Hulse et al., 2008). Each von Frey hair tested was applied a total of 5 times to each hind paw and the number of times an animal removed the paw from each stimulus was counted. The proportion of times that the animal withdrew from each stimulus was plotted against the breaking force, and the withdrawal threshold determined from the resultant stimulus response curve (the gram force at which paw removal occurred at 50% of the stimulations).

### Hargreaves test for thermal hyperalgesia

Thermal hyperalgesia was measured using a radiant heat source directed against the plantar surface of the hind paws, through the Perspex floor of the testing chamber (Hargreaves et al., 1988), and the latency to withdrawal was measured. The stimulus intensity was determined at the beginning of each experimental series, to give a control withdrawal latency of ~ 10 s, and this intensity was subsequently used for each subsequent testing session for that experimental group. A maximum latency duration of 30 s was used to prevent tissue damage/sensitization to intense sustained stimulation. The mean withdrawal latency was determined from three repeated stimulations at an inter-stimulus interval of at least 5 min.

### Model of neuropathic pain — partial saphenous nerve injury

24 mice and 18 rats underwent surgical partial saphenous nerve injury (PSNI) as previously described (Hulse et al., 2008, Walczak et al., 2005) under isofluorane anesthesia (2–3% in O_2_). A ~ 1 cm incision was made in the inguinal fossa region of the right hind leg. 50% of the saphenous nerve was tightly ligated using a size 6.0 sterile silk suture and the wound was closed with size 4.0 sterile silk suture. Sham-operated animals (n = 5) underwent anesthesia and surgery involving solely an incision in the inguinal fossa region of the right hind limb.

### Electrophysiological recording of identified primary afferents in the saphenous nerve

Teased fiber electrophysiology from the saphenous nerve was carried out in 44 adult male Wistar rats (250–300 g). Properties of isolated afferents in terminally anesthetized rats (sodium pentobarbital ~ 20 mg/kg/h) and the effects of local injection of compounds into the receptive fields were determined (see section Pharmacological treatments for further information), as previously described (Dunham et al., 2008, Hulse et al., 2010b). Fine nerve filaments were dissected from the main trunk of the nerve cut centrally and differential recordings were made using bipolar platinum wire recording electrodes. Primary afferents were identified in the filaments using mechanical and/or electrical search stimulation of identified receptive fields located in the dorsomedial region of the right hind paw, the area innervated by the saphenous nerve in the rat. Filaments usually contained a single identified afferent (unit), but up to 3 units could be studied in the same filament provided the receptive fields were distinguishable. Action potentials from each fiber could be distinguished individually by offline action potential recognition and sorting. Data capture was through a micro 1401-3 (Cambridge Electronic Design) and offline action potential sorting and analysis was carried out on Spike 2 version 7 (CED).

Identified units were characterized according to their conduction velocity (CV) and response to mechanical stimulation of the receptive field. Units that could not be activated by peripheral mechanical stimulation were not studied further. Monopolar electrical stimulation was applied to the receptive field (up to 100 V, 0.5 ms duration) and 3 reproducible action potential latencies were required to calculate the conduction velocity (CV). Following CV measurement, any ongoing activity (action potential firing) was recorded for 100 s. Note that under normal conditions, the majority of afferents in the saphenous nerve do not show significant ongoing activity, as there are no muscle spindles, and very few cooling afferents in this largely cutaneous sensory nerve. Ongoing activity was defined as firing > 0.1 impulses/s occurring without any obvious initiating factor. During the period of recording of ongoing activity, no further stimulation of the receptive field was applied.

Mechanical thresholds were determined as the lowest von Frey hair applied that elicited a robust (> 3 action potentials) reproducible response (Dina et al., 2004, Dunham et al., 2008, Koltzenburg et al., 1999a, Lynn and Carpenter, 1982). Responses to light brush with a paintbrush and to a series of von Frey hairs were then recorded. Primary afferents with a CV less than 1 m·s^− 1^ were classified as C fibers, based on compound action potentials recorded in the same preparation in animals of a similar weight, sex and age (Dunham et al., 2008). Afferents that were not brush sensitive, with von Frey thresholds > 1 g were classified as nociceptors (Dunham et al., 2008, Lynn and Carpenter, 1982); C fiber nociceptors were those that met these criteria and had CV < 1 m·s^− 1^. Ongoing activity was outlined as those units with greater than 0.1 Hz (Shim et al., 2005).

#### Methodological note

It should be noted that hand-held von Frey hairs give an approximation of the mechanical thresholds of primary afferent units as application of a range of hairs exerts incremental, discrete forces rather than a continuous force on the receptive field. As von Frey hairs were used for behavioral tests, comparable methods of single neuronal activation were used. Single afferent mechanical thresholds are typically lower than behavioral withdrawal thresholds, as withdrawal reflexes require summation of input from multiple high threshold nociceptive afferents for activation.

### Intracellular calcium measurements in primary dorsal root ganglion cells

DRG were dissected from adult Wistar rats, dissociated, and cultured as previously described (Wong et al., 2006). For TRPV1 experiments, following overnight pretreatment with VEGF-A isoforms in media or media alone, primary DRG cultures were pre-incubated with 100 μl of Ca^2 +^-sensitive dye (Fluo-4 direct) at 37 °C for 1 h during which time concentrated capsaicin agonist solutions at 6 × the final concentrations used were prepared and preheated to 37 °C. Fluorescence recordings were performed row by row. First the baseline fluorescence at 488 nm was read, then 20 μl capsaicin solution was added to the dye to achieve the final capsaicin concentration, and the fluorescence read within 45 s and then repeatedly every 10 s for ~ 4 min on a Wallac 1420 Victor 3™ multi-label reader (PerkinElmer Inc.). The change in fluorescence over baseline was determined for each recorded time point. Each capsaicin concentration was tested in multiple replicates (3–7). Some cultures were fixed, and stained for VEGFR2 (Cell Signaling, rabbit mAb 55B11).

### Capsaicin-evoked currents in primary dorsal root ganglion cells

Primary DRG cultures were prepared as above, and grown on glass coverslips coated with poly-l-lysine and laminin. After 3–5 days coverslips were mounted into a recording chamber and visualized using Olympus BX50WI microscope (Olympus, UK) using a 60× water immersion objective. Cells were chosen for study based on cell diameter (all < 30 μm diameter). Cells were continually perfused 2–3 ml/min with an extracellular solution containing (mM): 145 NaCl, 5 KCl, 0.5 CaCl, 2 MgCl_2_, 10 HEPES, and 10 d-glucose, pH was adjusted to 7.4 at 310–320 mOsm. Low calcium solution was used to reduce any calcium dependent desensitization of the current. Cells were patch-clamped in the whole-cell configuration and held at − 80 mV at room temperature (18–22 °C). Patch pipettes were pulled from soda glass (Harvard Apparatus, UK) to a resistance of 3–4 mΩ and the tips were coated in surf wax (Mr Zoggs) to reduce the capacitive transient. The pipette solution contained (mM): KCl 140, NaCl 5, EGTA 5, MgCl_2_ 2, HEPES 10, adjusted to pH 7.2 with KOH. Puff pipettes pulled from soda glass with resistances of 3–4 M were filled with 500 μM capsaicin in external solution (stock solution 10 mM dissolved in 10% DMSO, 10% Tween-80, 80% saline and diluted to working concentration in external solution) and positioned between 20 and 30 μm from the cell. A Pneumatic Picopump PV800 (WPI, Hertfordshire, UK) was used to apply a two second puff at 10 psi. Inward currents in response to the puff application were recorded using an axon 200B amplifier and pClamp 9 software (Axon Instruments, CA, USA) with a sampling rate 20 kHz and filtering at 5 kHz filter. The peak of the inward currents during puff application were measured using Clampfit 9 (Axon instruments).

### TRPV1 immunoprecipitation and Western blotting

Immortalized rat embryonic DRG cells, that represent largely nociceptive neurons expressing TRPV1 and NGF receptors (50B11) (Chen et al., 2007) were grown in Neurobasal medium (Invitrogen) supplemented with B27 (Invitrogen), 10% FBS, 0.5 mM l-glutamine and an additional 2.2% glucose in 6 well plates. These neurons were used as an alternative to primary culture, to generate the amounts of protein required for phospho-immunoprecipitation, as required by the principles of the ‘3Rs’ (reduction, replacement, refinement) under UK and EU legislation. Upon reaching 80% confluence cells were differentiated for 24 h with 75 μg/mL forskolin and 0.1 nM NGF-2.5S, and then treated overnight (~ 16 h) with VEGF-A proteins (2.5 nM), NGF-2.5S (4 nM) or vehicle (PBS). Following treatment, cell lysate protein, extracted in the presence of phenylmethylsulfonyl fluoride and proteinase inhibitors, was subjected to immunoprecipitation using a TRPV1 antibody (Abcam, rabbit pAb, ab10296) and Millipore PureProteome™ Protein A Magnetic Bead System. The manufacturer's direct immunoprecipitation method was followed using 2 μg/mL antibody and 50 μg protein lysate. The eluates were separated on 10% SDS-PAGE gels blotted on PVDF membrane by wet transfer and incubated overnight with 2 μg/mL phospho-serine mouse mAb (Millipore, 4A4). After secondary antibody incubation (goat pAb HRP-anti-mouse IgG) blots were developed using Pierce ECL SuperSignal Femto reagent. Blots were stripped and re-probed using the aforementioned TRPV1 rabbit pAb. Cell lysate protein not subjected to immunoprecipitation was separated and blotted as above and TRPV1 levels detected. This indicated the TRPV1 input level into the immunoprecipitation.

### VEGF-A isoform and VEGFR2 expression studies — immunofluorescence, ELISA and qRT-PCR

Total VEGF-A and VEGF-A_165_b were detected using validated, commercially available antibodies (Santa Cruz A-20, and AbCam MVRL56/1 respectively). The VEGF-A_165_b antibody detects the unique C-terminal of the alternatively spliced VEGF-A_xxx_b family (Woolard et al., 2004). VEGF-A_165_a levels were determined by subtraction of VEGF-A_165_b levels from total VEGF-A.

For immunofluorescence in rat DRG, animals were terminally anesthetized with sodium pentobarbital (60 mg/ml) and perfuse fixed with 4% paraformaldehyde (PFA). Ipsi- and contralateral lumbar dorsal root ganglia were removed. DRG were placed into PFA for 4 h and then cryoprotected in 30% sucrose solution overnight. 8 μM cryosections were cut from OCT embedded DRG and thaw mounted onto subbed slides. Sections were blocked in 10% fetal calf serum/5% bovine serum albumin/0.2% Triton × 100 in PBS for 2 h at room temperature. Primary antibodies were used at the concentrations below diluted in the blocking solution and incubated overnight at 4 °C. Prior to secondary antibody incubations sections were washed extensively with PBS. Sections were incubated in secondary antibodies diluted in 0.2% Triton × 100 in PBS for 2 h at room temperature. Streptavidin amplification was performed as per manufacturer's instructions (Vector, UK). Primary antibody concentrations: TrkA, 1 μg/ml; 56/1, 12 μg/ml; α-phospho-tyr1175 VEGFR2 and VEGFR2 (1 in 500 dilution). Secondary antibodies: biotinylated anti-rabbit (Jackson Immune Research, diluted 1 in 500). Alexa Fluor 488 or 594 were used at 1 in 1000 dilution. α-goat Alexa Fluor 594 (Invitrogen, UK). Hoechst (Sigma Aldrich) and goat SRSF1 (2 μg/ml, Santa Cruz). Images were visualized using a fluorescent microscope and captured before quantification.

VEGF-A_165_b splice variant levels were measured as a proportion of total VEGF-A_xxx_ expression using sandwich ELISA (R&D systems Duoset VEGF ELISA DY-293) using N-terminal goat anti-human VEGF antibody against pan-VEGF (i.e. all the isoforms), and mouse anti-human antibody against VEGF-A_165_b (R&D systems), as previously described (Gammons et al., 2013).

Real-time qRT-PCR. 2 μg of DNase-digested total RNA was reverse transcribed using oligo (dT15) and random primers (Promega, UK). Real-time PCR was performed on a ABI 7000 thermocycler using ABsolute QPCR SYBR green mix (Thermo Scientific, UK) and 1 μM primers specific for VEGF-A_165_a, (forward — exon 7/8a: 5′-GTTCAGAGCGGAGAAAGCAT-3′; reverse 5′- TCACATCTGCAAGTACGTTCG-3′) and total VEGF-A (forward — exon 2/3: 5′-GGAGGGCAGAATCATCACGAAG-3′; reverse 5′-CACACAGGATGGCTTGAAGATG-3′) (Nowak et al., 2010, Woolard et al., 2004), and for the housekeeping genes (18S ribosomal, GAPDH and Microglobulin) (see (Amin et al., 2011) for primer sequences). Cycling conditions were: denaturation at 95 °C for 10 min, then 95 °C for 15 s, and 55 °C for 30 s for 40 cycles. RNA levels were estimated by (VEGF-A_165_a/VEGF-A_total_) = E^−(CtVEGF165)^/E^−(CtVEGFtotal)^ where E is the efficiency of the qPCR reaction for the primer pair used, or for expression relative to housekeeping genes, V = 2^−(ΔCt)^ where ΔCt is the difference between the cycle thresholds for VEGF-A and housekeeping gene. Values are expressed relative to saline treated tissues.

#### Experimental note

The presence and function of endogenous VEGF-A_165_b has been demonstrated using specific siRNA knockdown, expression in multiple cells, tissues and pathological conditions using rigorous controlled techniques (Harper and Bates, 2008) and the importance of VEGF-A_165_b in pathological conditions has recently been highlighted by its ability to predict response to bevacizumab in the registration trial in colorectal cancer (Bates et al., 2012). A recent study highlighted the importance of using appropriate controls to avoid artifactual detection of VEGF-A_xxx_b isoforms (Harris et al., 2012) in rodents. A further study (Bates et al., 2013) demonstrates clearly the importance of positive and negative controls, for example for effective PCR amplification, to eliminate non-specific antibody binding to mouse IgG and to prevent mispriming. To avoid possibilities of misinterpretation of artifactual amplification of products we used pro-angiogenic isoform specific primers (against exon 8a) and total VEGF-A primers to determine the effect of splicing inhibitors on VEGF-A splicing.

### Pharmacological treatments

A) VEGF neutralization and VEGF receptor block. Mechanical and thermal nociceptive behaviors were determined in adult mice before and 2 days after intraperitoneal (i.p.) injection with 6 μg/g bodyweight mouse G6-31 antibody (n = 5, both hind paws used as replicates) or vehicle (saline, n = 6).
  VEGFR2 tyrosine kinase inhibitors PTK787 and ZM323881 were given systemically to rats by a single i.p. injection (PTK787: 30 mg/kg, 30 μg/g) or locally into the hind paw (rats and mice: ZM323881: 10 μl containing 100 nM). Vehicle (PTK787, saline; ZM323881, 0.001% DMSO in saline) was injected by the same route (n = 6/group). ZM323881 was given under brief isofluorane anesthesia (2–3% in O2). Nociceptive behavior was tested before and after treatment (PTK787 2 h; ZM323881 20–40 min).
B) Effect of exogenous VEGF-A_165_a and VEGF-A_165_b in control animals and after peripheral nerve injury:
  i) Nociceptive behaviors — normal. Saline (200 μl), VEGF-A_165_a (8 ng/g body weight), VEGF-A_165_b (8 ng/g), VEGF-A_165_b (20 ng/g), VEGF-A_121_a (8 ng/g) and VEGF-A_159_ (8 ng/g) were injected i.p biweekly (n = 5 per group) and nociceptive behaviors assessed before and after administration for a period of 5 days (experiments performed in mice).
  ii) Nociceptive behaviors — PSNI. After surgery for peripheral nerve injury, groups of mice received one of the following biweekly: VEGF-A_165_a (n = 6, 8 ng/g i.p. (Zheng et al., 2007)); VEGF-A_165_b (n = 6, 20 ng/g) or PBS vehicle (n = 16, 200 μl). Injections were given immediately after PSNI surgery, and after behavioral test sessions on days 3 and 7. Sham-operated controls (n = 5) received i.p. vehicle at the same times. Nociceptive testing was performed on days 1, 3, 5, 7 & 10 after PSNI.
  iii) Primary afferent properties.Afferents were isolated and characterized as described above. The effect of rhVEGF-A_165_a injected locally into the receptive field (10 μl; 2.5 nM) on afferent activity was determined by a change in ongoing activity. In these experiments we determined that ~ 50% of identified nociceptive cutaneous afferents responded to VEGF-A_165_a with an increase in spontaneous action potential firing over a period of 1 h (Fig. 3E). In subsequent experiments, nociceptive afferents responding to VEGF-A_165_a were identified by this ongoing activity. Afferents were characterized by CV and mechanical threshold, and mechanical stimulus-evoked responses were recorded. VEGF-A was then injected locally, and these properties were recorded at set intervals for 60 min. Post-hoc analysis of mechanical threshold, mechanically evoked and ongoing activity recorded at 5, 30 and 60 min after VEGF-A administration was then done for those units that developed ongoing firing by 60 min.
C) Effect of SRPK1 inhibition in normal skin (mice) and after nerve injury (rats) on nociceptive behavior and VEGF-A isoform expression: SRPIN340 (10 μl; 10 μM), an inhibitor of SR protein kinases SRPK1 and 2 (Fukuhara et al., 2006) that are responsible for splicing control of VEGF-A isoforms (Nowak et al., 2010) or vehicle (saline) was injected into the plantar surface of one hind paw under brief isofluorane anesthesia (2–3% in O_2_). Nociceptive behavior was tested before and after injection (n = 6/group). Animals were killed by anesthetic overdose, and the plantar skin at the site of injection removed. Total VEGF-A and VEGF-A_165_b mRNA expression was determined at the site of SRPIN340 or saline injection by quantitative (q) PCR.
  To investigate the effect of inhibition of splicing in the injured nerve, 8 rats underwent PSNI surgery and were treated with the SR protein kinase (SRPK1/2) inhibitor, SRPIN340 (Nowak et al., 2010) or vehicle (saline). SRPIN340 (10 μM) was incorporated into a sterile gel consisting of (2% hyproxymethylcellulose, 0.2% tyloxapol, 3.4% dextrose, 0.025% ethylenediaminetetraacetic acid (EDTA), 0.0006% benzalkonium chloride) and applied to the nerve in the area of the tight ligation (n = 4); gel without SRPIN340 was used in control animals (n = 4). Nociceptive testing was performed on days 1 and 2 after PSNI. Animals were killed by anesthetic overdose and the saphenous nerves and L3/L4 DRGs removed. VEGF-A splice variant mRNA expression was determined at the site of injury by qRT-PCR for total VEGF-A and VEGF-A_165_a.
D) Interactions between VEGF-A and TRPV1:
  i) Effect of pharmacological TRPV1 receptor blockade (mice): Mechanical nociceptive behavior was determined every other day for 5 days. VEGF-A and SB366791 (500 μg/kg) or vehicle were given via systemic (i.p.) injection on days 1 and 3 immediately after behavioral testing (n = 3/group, both hind limbs tested and treated as replicates). To exclude an effect of pharmacological inhibition on central rather than peripheral TRPV1, 2.5 nM VEGF-A_165_a was injected subcutaneously into the plantar surface of the hindpaw was with either PBS or 1 μM SB366791 (n = 5 per group) followed by behavioral testing.
  ii) VEGF-A-mediated TRPV1 sensitization (rats): To determine the effect of VEGF-A isoforms administered together with a non-sensitizing concentration of TRPV1 agonist in identified primary afferents, a bolus of 10 μM capsaicin was injected through an intra-femoral arterial cannula inserted in the mid-thigh in the opposite hind limb to the recordings, with the tip advanced to the bifurcation of the descending aorta. This allowed close arterial delivery of capsaicin (100 μl; 10 μM washed in with 400 μl saline) to the peripheral afferent receptors. The effects of VEGF-A_165_a and VEGF-A_165_b on TRPV1 agonist responses were investigated using close arterial injection of capsaicin combined with local (subcutaneous) injection of 2.5 nM VEGF-A_165_a, 2.5 nM VEGF-A_165_b or both together. When VEGF-A_165_b was used, it was then followed by VEGF-A_165_a to confirm VEGF receptor responses in the afferents studied, as described above.

Numbers of afferents included in the experiments were: ongoing and mechanically evoked activity — saline vehicle n = 12, VEGF-A_165_a n = 7, and VEGF-A_165_b n = 5; mechanical activation threshold — VEGF-A_165_a, saline n = 7, VEGF-A_165_b n = 5; capsaicin sensitization — baseline n = 16, VEGF-A_165_a n = 8, VEGF-A_165_b n = 8, VEGF-A_165_a + VEGF-A_165_b n = 7.

Experimental note: This concentration of capsaicin delivered by close arterial injection does not result in sensitization or desensitization of the TRPV1 receptors to agonist stimulation on repeated injection (Dunham, 2008, Dunham et al., 2008). A low capsaicin concentration to avoid possible desensitization of TRPV1 in the presence of a further sensitizing agent, as we hypothesized VEGF-A_165_a to be. Capsaicin injection resulted in a short burst of action potentials that confirmed access of the agonist to the afferent receptor terminals. It should be noted that the effective concentration of capsaicin at the primary afferent terminals when delivered by this method is approximately 1000 fold lower than that injected as a result of dilution in hind limb blood volume, and tissue penetration (Dunham, 2008).

#### Statistical analyses

The majority of data sets was Gaussian in nature and therefore met the requirements for parametric analyses; in a small number of cases, data sets were log transformed to render them Gaussian prior to analysis (e.g. withdrawal thresholds). Multiple groups were compared using one or two way ANOVA followed by post-hoc Bonferroni tests where appropriate, and where Gaussian assumptions were not met or log transformation did not render the samples Gaussian, non-parametric tests were used, in which case multiple groups were compared with Kruskal–Wallis or Friedman's tests followed by post-hoc Dunn's tests. Two group tests were 2 tailed Student's t-tests with Welch's correction where necessary for unequal variance, or Mann Whitney U tests for non-parametric data. Numbers of DRG neurons with TRPV1-activated currents were compared using Fisher's exact test.

## Results

### VEGF-A splice isoforms differentially affect pain behaviors, through direct VEGFR2-mediated effects on primary sensory nociceptive neurons

**Fig. 2:**
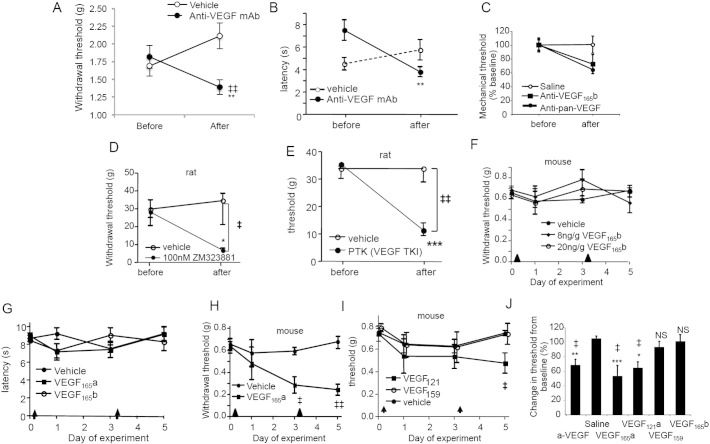
VEGF-A isoforms differentially affect pain depending on VEGFR2 activation. A. Intraperitoneal injection of 6 μg/g anti-VEGF-A antibody induced significant mechanical allodynia in mice (n = 5; vehicle n = 6). B. Systemic injection of anti-pan-VEGF-A antibody (6 μg/g) but not vehicle lowered thermal nociceptive withdrawal latency. C. Mechanical allodynia was reproduced by an anti-VEGF-A_165_b antibody (n = 6), shown normalized to the data from  panel A. D. Local blockade of VEGFR2 with 100 nM ZM323881 (specific for VEGFR2) resulted in mechanical allodynia (n = 6/group). E. Systemic injection of PTK787 (30 μg/g) significantly reduced mechanical withdrawal threshold in naïve rats compared to vehicle (saline, n = 6/group). F. rhVEGF-A_165_b (8 ng/g or 20 ng/g) was not painful in normal animals (n = 5/group). Arrowheads denote times of drug administration. G. Neither rhVEGF-A_165_a nor rhVEGF-A_165_b (both 8 ng/g bodyweight) affected thermal hyperalgesia in naïve mice compared to vehicle (saline, n = 5/group). H. rhVEGF-A_165_a (8 ng/g) induced mechanical allodynia. I. rhVEGF-A_121_a administration caused mechanical allodynia whereas rVEGF-A_159_ did not (n = 5/group). J. Comparison of the effects of different VEGF-A isoforms shows that rhVEGF-A_xxx_a-evoked allodynia is mediated by the C-terminal 6 amino acids. * = p < 0.05, ** = p < 0.01, *** = p < 0.001 compared with baseline measurements within the same group, ‡ = p < 0.05, ‡‡ = p < 0.01, ‡‡‡ = p < 0.001, between groups, NS = not significantly different. Mean ± SEM for mouse behavior, and median ± IQR for rat behavior.

Systemic delivery of anti-mouse VEGF antibody acutely sensitized animals to mechanical (Fig. 2A) and thermal (Fig. 2B) stimulation. Neutralization of VEGF-A_165_b (Fig. 2C), and inhibition of VEGF receptor-2 (VEGFR2) by selective (ZM323881, Fig. 2D) and specific (PTK787, Fig. 2E) inhibitors also produced sensitization. Systemic recombinant human (rh)VEGF-A_165_b (up to 20 ng/g bodyweight, i.p.) had no effect on mechanical (Fig. 2F), or thermal (Fig. 2G) nociceptive behavior, whereas rhVEGF-A_165_a (8 ng/g bodyweight, i.p.) sensitized to mechanical (Fig. 2H) but not thermal stimuli (Fig. 2G). rhVEGF-A_121_a, which has the same C-terminal six amino acid sequence as the VEGF-A_xxx_a family but reduced affinity for neuropilin-1 (NP-1) also resulted in mechanical sensitization (Fig. 2I). rVEGF-A_159_, which lacks the six C terminal amino acids (Cebe Suarez et al., 2006), had no effect on pain (Fig. 2I), showing that the mechanism through which VEGF-A_165_a and VEGF-A_121_a enhance pain is C-terminal sequence dependent (summarized in Fig. 2J) .

**Fig. 3:**
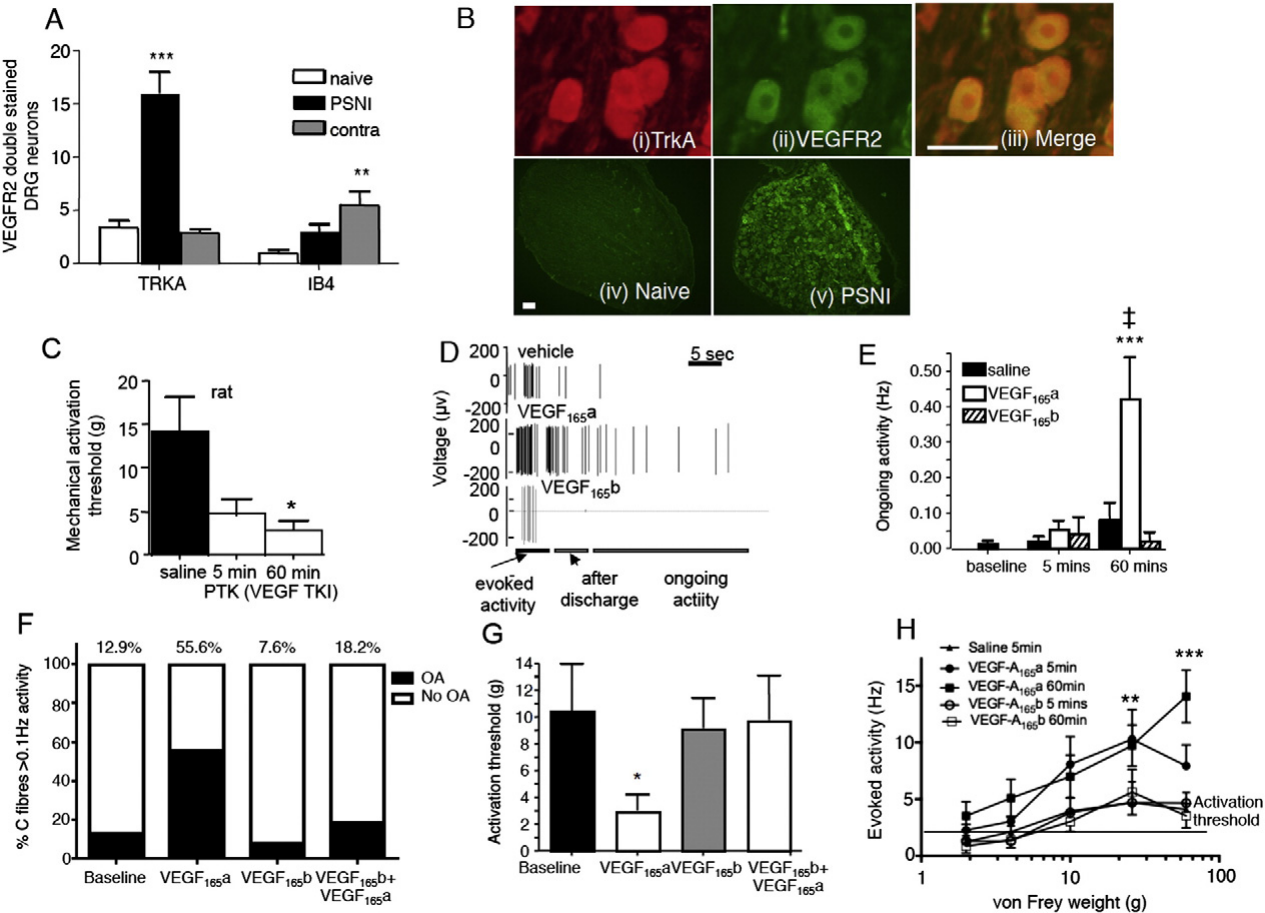
Effects of rhVEGF-A isoforms on primary afferent nociceptors. A. VEGFR2 is expressed in nociceptive sensory neurons as determined by double-labeling with the nociceptive markers TrkA (high affinity nerve growth factor receptor) and isolectin B4 (IB4). VEGFR2 expression is upregulated in TrkA + ve nociceptors ipsilateral, and in IB4-binding nociceptors contralateral, to partial saphenous nerve injury (PSNI). B. Photomicrographs of (i) TrkA positive DRG neurons, (ii) VEGFR2 positive neurons and (iii) merged images of (i) and (ii) showing TrkA and VEGFR2 colocalization (scale bar = 50 μm). (iv) Expression of VEGR2 in DRG neurons is much lower in naïve rat DRG compared to (v) animals with PSNI (scale bar = 100 μm). C. Endogenous VEGF-A moderates nociceptor sensitivity, as when VEGFR2 is inhibited by PTK787 mechanical activation threshold of individual nociceptors is reduced within 5 min and over the next 60 min, indicating sensitization. D. Digitized data trace showing the effect of vehicle (saline), VEGF-A_165_a and VEGF-A_165_b on mechanically evoked activity at 5 min, after discharge and ongoing activity in a single afferent nociceptor. rhVEGF-A_165_a sensitized afferents to mechanical stimulation, enhancing after discharge and ongoing activity. Vertical lines are time-compressed action potentials. E. Increased spontaneous ongoing activity was evoked by rhVEGF-A_165_a but not rhVEGF-A_165_b in ~ 50% of mechanonociceptive afferents in rats. (Saline vehicle n = 12, VEGF-A_165_a n = 15, VEGF-A_165_b n = 5). Graphs include data from all neurons, including those in which properties did not change in response to VEGF-A. F. VEGF-A_165_a led to increased ongoing activity in 56% of nociceptive C fibers (OA > 0.1 Hz (Shim et al., 2005)). VEGF-A_165_b did not alter the degree of ongoing activity or number of C fibers that demonstrated ongoing activity, and in addition blocked VEGF-A_165_a-induced ongoing activity. G. rhVEGF-A_165_a reduced primary afferent mechanical threshold 60 min after rhVEGF-A_165_a injection. This was not seen for rhVEGF-A_165_b, and was blocked by its co-administration. H rhVEGF-A_165_a increased primary afferent activity in response to stimulation at suprathreshold force, 5 and 60 min after the injection of rhVEGF-A_165_a, whereas saline and rhVEGF-A_165_b had no effect. * = p < 0.05, ** = p < 0.01, *** = p < 0.001 compared with saline, mean ± SEM.

We then determined the neuronal mechanism through which systemic rhVEGF-A_165_a might alter nociceptive behavior. VEGFR2 protein was detected in DRG neurons (Figs. 3A & B) as previously described (Lin et al., 2010, Sondell et al., 2000) by immunofluorescence in proportions of both TrkA- and isolectin B4, nociceptive neurons (Fig. 3A), with increased expression following traumatic nerve injury. Inhibition of VEGFR2 (locally applied PTK787 to the receptive field) directly sensitized nociceptors to mechanical stimulation (Fig. 3C). As endogenous VEGF-A isoforms can exert potent vascular effects, we also determined whether VEGFR2 inhibitors PTK787 and ZM323881 overtly affected local blood flow. Neither receptor blocker resulted in any reduction in local blood flow as measured by laser Doppler flowmetry, or in skin temperature, in contrast to local adrenaline injection used as a positive control (data not shown). To determine the roles of VEGF-A isoforms on sensory afferents, effects on nociceptors were determined before and after injection of vehicle, VEGF-A_165_a or VEGF-A_165_b (Fig. 3D). Injection of rhVEGF-A_165_a, but not rhVEGF-A_165_b into individual characterized sensory neuronal receptive fields resulted in the initiation of spontaneous ongoing firing (Fig. 3E) in 56% of mechano-sensitive primary afferent nociceptors tested (Fig. 3F), indicating expression of *functional* VEGF receptors in a large proportion of the sampled afferents. The proportion of neurons responding to VEGF-A (> 50%) was significantly higher than the proportion of VEGFR2 positive IB4/TrkA + ve neurons — posited to be nociceptors (Fang et al., 2005, Fang et al., 2006). There are a number of explanations for this including: unconscious bias in our search strategy for afferents (mechanosensitive, C-fiber nociceptors) leading to an over-representation of afferents expressing VEGFR2; detection of protein by immunofluorescence underestimating the degree to which *functional* VEGFR are found on sensory neurons; or a higher proportion of VEGF sensitive nociceptors in the paw than in other regions through which L4 DRG neurons receive input. As the characteristics and distribution of VEGF sensitive afferents are not fully known in any species, any bias was unavoidable, and only came to light in post-hoc analysis.

Those neurons that developed ongoing firing after VEGF-A_165_a administration also became more sensitive to mechanical stimulation after 5 min (Fig. 3H, evoked activity at 5 and 60 min after rhVEGF-A_165_a, main effect of drug p < 0.0001), had lowered mechanical activation thresholds at 5 min (thresholds were saline: 6 (9) g (median (range)); rhVEGF-A_165_a: 1.5 (3.9) g; rhVEGF-A_165_b: 4 (14.4) g, ANOVA p = 0.08) and 60 min (Fig. 3G) and increased after discharge post-stimulus at 5 (Fig. 3D) and 60 min (not shown), indicating VEGF-A_165_a mediated peripheral neuronal sensitization that would translate into increased sensitivity to painful mechanical stimulation. Conversely, VEGF-A_165_b does not lead to sensitization of nociceptor activity (Figs. 3E, F, G and H) and importantly, completely abolished VEGF-A_165_a induced nociceptor hyperexcitability in all instances (Figs. 3E, F, G and H). Thus both VEGFR2 inhibition and VEGFR2 activation by rhVEGF-A_165_a enhance nociception by sensitization of peripheral mechanosensitive nociceptors.

**Fig. 4:**
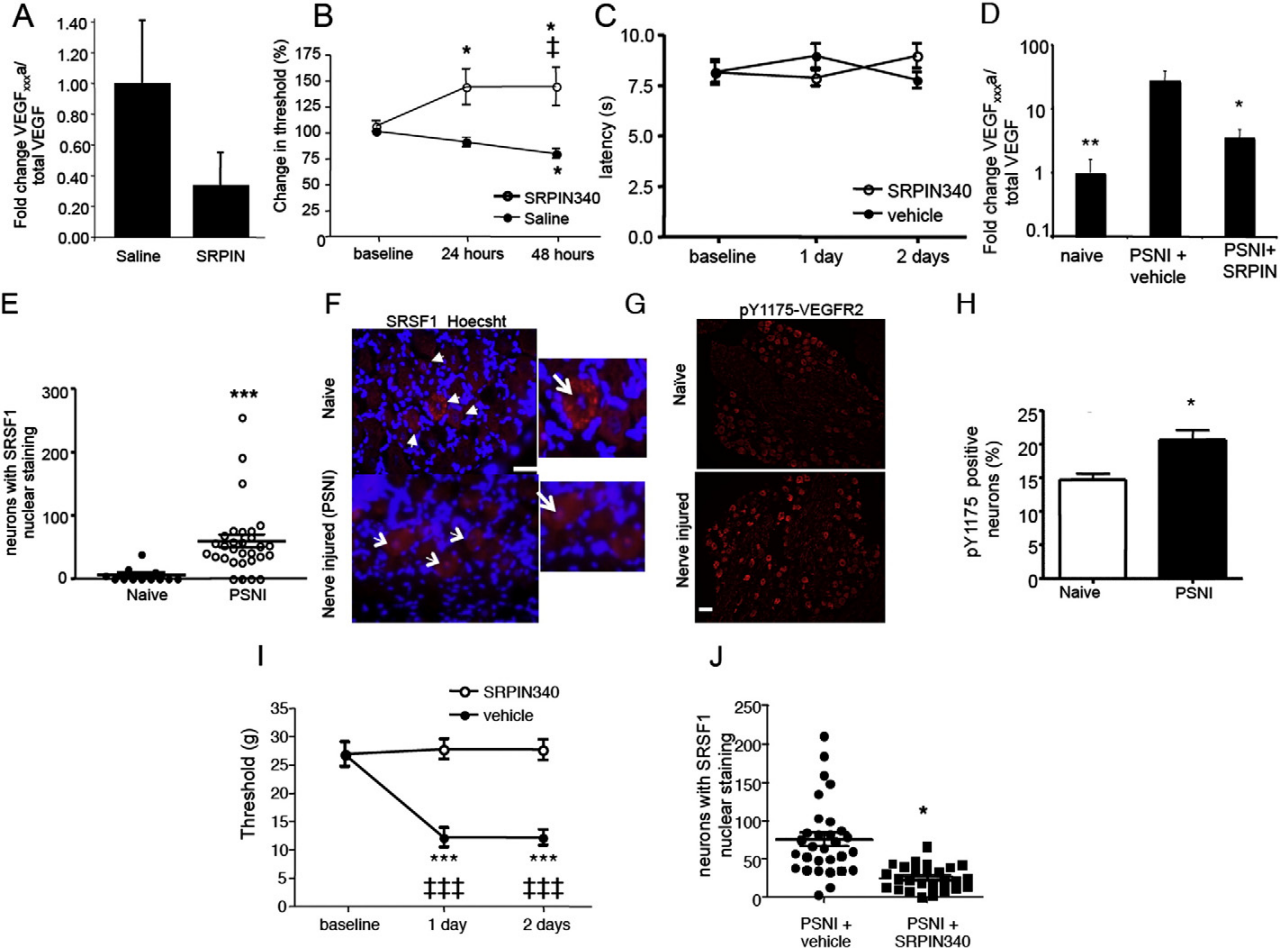
Splicing inhibitors that shift the balance of endogenous VEGF-A towards an excess of VEGF-A_xxx_b isoforms are anti-nociceptive in normal and nerve injured rats. A. Intraplantar injection of SRPK1 inhibitor SRPIN340 reduced the amount of VEGF-A_165_a mRNA as a proportion of the total VEGF-A mRNA in plantar skin compared to vehicle (saline). B. SRPK inhibition raised mechanical withdrawal thresholds i.e. resulted in hypoalgesia, in mice. C. SRPIN340 did not alter thermal withdrawal latencies. D. VEGF-A_xxx_a expression increased as a proportion of total VEGF-A after PSNI. This increase was inhibited by SRPK inhibition. E. Nuclear localization of SRSF1, indicative of SRPK1 activity, is increased in L3/4 DRG neurons following PSNI. F. SRSF1 expression (red) in the cytoplasm of naïve rat DRG sensory neurons (scale bar 50 μm) and SRSF1 expression in the nucleus (stained blue with Hoechst) of rat DRG sensory neurons following PSNI. Note blue staining of nuclei in naïve rats, but purple in PSNI (inset, arrow). G. Phosphorylated (p)Y1175-VEGFR2 (red) staining in naïve and nerve injured mice. H. The number of pY1175-VEGFR2 positive DRG neurons increased after PSNI (*p = 0.019). I. SRPIN340 prevented PSNI-induced mechanical allodynia. J. SRPIN340 reduced SRSF1 activation in DRG containing injured neurons 2 days after nerve injury. ‡, ‡‡‡, p < 0.05, 0.001 respectively compared to baseline; *, *** = p < 0.05, 0.001 respectively compared to other groups.

Alternative splicing of pre-mRNA to VEGF-A_xxx_a rather than VEGF-A_xxx_b is controlled by the constitutively active serine–arginine protein kinase SRPK1 (Nowak et al., 2010), leading to activation of the splicing factor SRSF1 and selection of the proximal splice site (Fig. 1). On activation, SRSF1 translocates to the nucleus, and therefore activation of SRSF1 and subsequent splice site choice can be assessed by the degree of nuclear localization (Ghosh and Adams, 2011). SRPK1 inhibition and block of SRSF1 function, thus enhancing distal splice site selection, results in an increased proportion of VEGF-A_xxx_b (Fig. 1). Subcutaneous injection of an SRPK inhibitor (SRPIN340) in normal rat hind paw switched splicing, reducing VEGF-A_165_a relative to total VEGF-A to 33% of control levels in skin (Fig. 4A). This was associated with a 50% increase in mechanical threshold (Fig. 4B), but no effect on thermal withdrawal latency (Fig. 4C). We then determined whether VEGF-A_xxx_a expression was altered in traumatic nerve injury (Hulse et al., 2008). After peripheral saphenous nerve injury (PSNI), there was a > 10 fold increase in the expression of VEGF-A_165_a mRNA (Fig. 4D) in the local environment at the site of injury. In the same animals, there was also increased nuclear localization of SRSF1 in the damaged L4 DRG (Fig. 4E, F), consistent with a switch in SRPK1 mediated splicing to VEGF-A_xxx_a in neurons. Staining of DRG for Y1175-phosphoVEGFR2 (Fig. 4G) demonstrated increased numbers of VEGFR2-pY1175 positive neurons (Figs. 4G, H), indicative of increased VEGFR2 activation in these neurons. SRPK inhibition by SRPIN340 as a depot at the site of nerve injury blocked the change in mechanical withdrawal threshold (Fig. 4I), with no effect on thermal withdrawal latencies (data not shown). It also blocked the increased expression of VEGF-A_165_a mRNA (Fig. 4D) and the SRSF1 activation in DRG neurons (Fig. 4J). In SRPIN340 treated animals there were no contralateral changes in either mechanical or thermal nociceptive behavior (not shown).

**Fig. 5:**
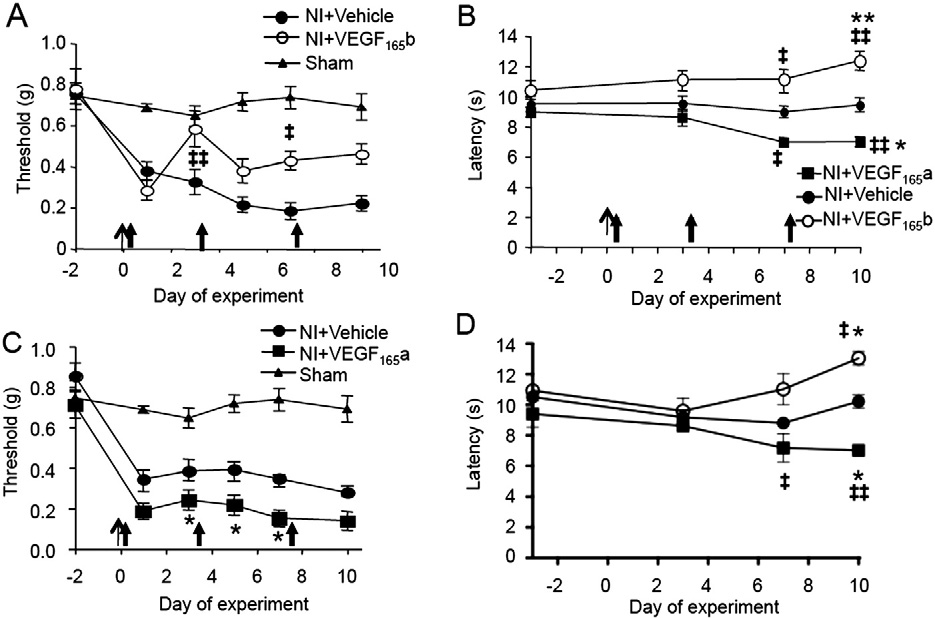
Exogenous VEGF-A_165_a exacerbates, and VEGF-A_165_b alleviates neuropathic pain. A. PSNI resulted in ipsilateral mechanical allodynia (NI + Vehicle) compared with sham and baseline. rhVEGF-A_165_b (20 ng/g) was anti-allodynic on days 3 (p < 0.001), 7 (p < 0.01) and 10 (p < 0.0001). Nerve injury on day 0, arrowheads denote drug injection. B. PSNI does not normally result in thermal hyperalgesia (NI + vehicle), but rhVEGF-A_165_a induced hyperalgesia (NI + VEGF-A_165_a) and rhVEGF-A_165_b hypoalgesia. C. rhVEGF-A_165_a (8 ng/g) enhanced ipsilateral mechanical allodynia (filled squares) compared to vehicle (filled circles). D. rhVEGF-A_165_a induced thermal hyperalgesia contralateral to PSNI. rhVEGF-A_165_b again resulted in hypoalgesia. ‡, ‡‡‡, p < 0.05, 0.001 respectively compared to baseline (not shown for mechanical thresholds for clarity as all significant); *, *** p < 0.05, 0.001 respectively compared to vehicle.

**Fig. 6:**
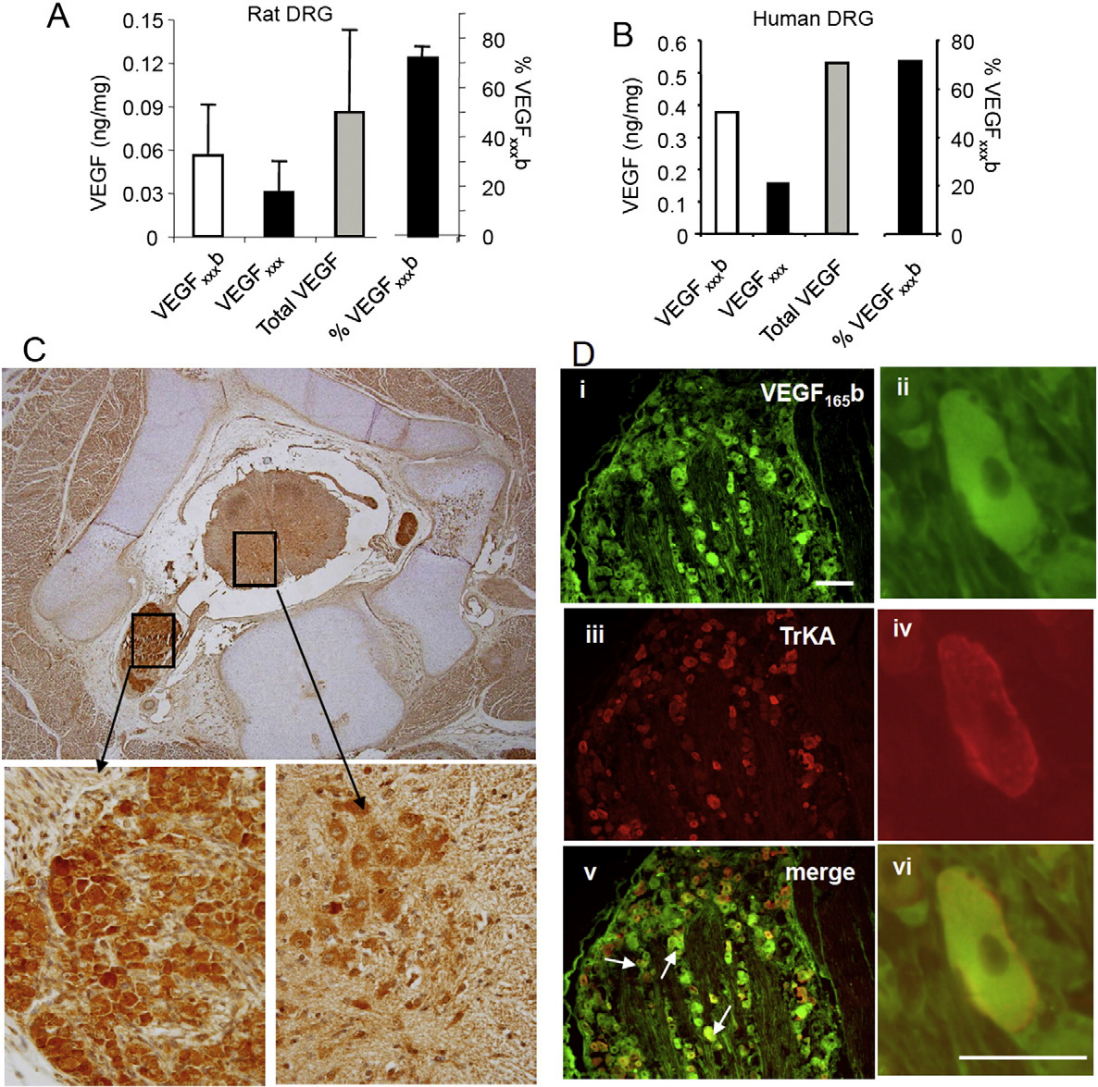
Expression of VEGF-A_165_a and VEGF-A_165_b in rat DRG. A. VEGF-A_165_b represents ~ 70% of total VEGF-A expression in DRG. B. In one human DRG VEGF-A_165_b represented a similar proportion of total VEGF-A expression to that seen in the rat. C. VEGF-A_165_b is expressed in neurons in embryonic human spinal cord and DRG. Higher magnification images are derived from the boxes in the top image and are left: DRG and right: spinal cord ventral horn. D. VEGF-A_165_b is expressed in a proportion of rat DRG neurons (Ai, iii, v), with overlap (arrows) with the nociceptive markers TrkA (Aii, iv, vi) and a small colocalization with IB4 (Aii, iv, vi). Scale bar = 75 μm. High power images of a single neuron showing colocalization of VEGF-A_165_b (green) and TrkA (red). Scale bar = 50 μm.

As nerve injury shifted the balance of VEGF-A isoforms towards VEGF-A_xxx_a, in both injured neurons and at the site of nerve injury, resulted in pro-nociception, and through blockade of this SPRK1–SRSF1 mediated switch with SRPIN340, VEGF_xxx_a mediated pro-nociceptive actions could be reversed, we hypothesized that altering the relative balance of VEGF-A isoforms with exogenous protein would have a similar effect. In contrast to normal animals (Fig. 2F), systemic rhVEGF-A_165_b treatment exerted anti-nociceptive effects on both mechanical (Fig. 5A) and thermal behavior (Fig. 5B) after PSNI, whereas rhVEGF-A_165_a was pro-nociceptive (Figs. 5B & C). Similar changes in thermal latencies but not in mechanical thresholds were also seen in the contralateral hindpaw (Fig. 5D), suggesting that central VEGF-A-dependent mechanisms may also contribute to changes in thermal nociception following nerve injury. It is possible that rhVEGF-A_165_b exerted little effect in uninjured animals because VEGF-A_165_b is the predominant VEGF-A isoform in both skin (Pritchard-Jones et al., 2007), and human and rat DRG neurons (~ 70% total, measured by ELISA, Figs. 6A & B), where it is expressed (Fig. 6C) in a proportion of TrkA-positive nociceptive neurons (Fig. 6D).

### VEGF-A isoforms affect pain by a TRPV1-dependent mechanism

**Fig. 7:**
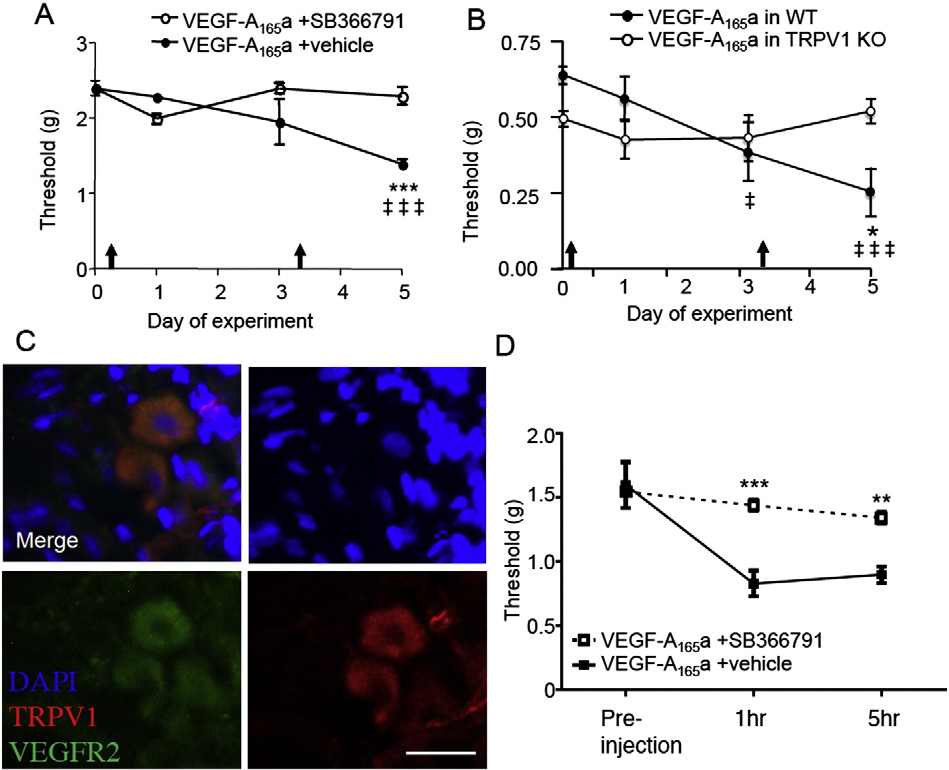
VEGF-A isoforms alter nociception in a TRPv1 dependent manner. A. Systemic TRPV1 antagonism with SB366791 in mice resulted in inhibition of rhVEGF-A_165_a-induced mechanical allodynia. Arrows denote time of drug administration. B. TRPV1 knockout mice did not develop rhVEGF-A_165_a-induced mechanical allodynia, in contrast to wild-type strain matched controls. C. TRPV1 was co-expressed with VEGFR2 in sensory dorsal root ganglia sensory neurons (scale bar = 20 μm). D. Local administration of VEGF_165_a + vehicle into the plantar hindpaw resulted in a reduction in mechanical withdrawal values, which was blocked by co-administration of the TRPV1 antagonist SB366791 (TRPV1 antagonist).

Sensitization through phosphorylation of the TRPV1 ‘capsaicin’ receptor is a common endpoint in the sensitization of many nociceptors to both thermal and mechanical stimulation in inflammation, and nerve injury (Levine and Alessandri-Haber, 2007). TRPV1 is a thermal (Caterina et al., 1997), not a mechano-transducer molecule, but TRPV1 agonists are well recognized to alter *both* thermal and mechanical thresholds in humans (Fluhr et al., 2009). TRPV1-expressing peripheral sensory nerves are mechanosensitive in addition to thermosensitive (Brenneis et al., 2013). There is substantial evidence of an involvement of TRPV1 in mechanical sensitization in visceral afferents (see references in Jones et al., 2005, Kiyatkin et al., 2013, Ravnefjord et al., 2009). Peripheral sensitization of afferents involving TRPV1-dependent mechanisms has also been reported in deep tissue afferents (Kelly et al., 2013, Lam et al., 2009), and importantly for these data, in skin, where TRPV1 sensitization by agonist, such as capsaicin, lowers mechanical thresholds and hence contributes to enhanced mechanonociception (Li et al., 2008, Ren et al., 2005, Ren et al., 2006). Systemic pharmacological antagonism (using SB366791 Fig. 7A) and TRPV1 knockout (Fig. 7B) both eliminated VEGF-A_165_a-mediated mechanical allodynia indicating that the mechanism of action of VEGF-A_165_a involves, at least in part, TRPV1. TRPV1 was colocalized with VEGFR2 in DRG neurons (Fig. 7C). Administration of locally applied VEGF-A_165_a to the plantar surface of the hindpaw led to mechanical hypersensitivity, which was blocked by local co-administration of the TRPV1 antagonist SB366791 (Fig. 7D), indicating peripheral TRPV1 in VEGF-A_165_a-induced mechanical hypersensitivity.

**Fig. 8:**
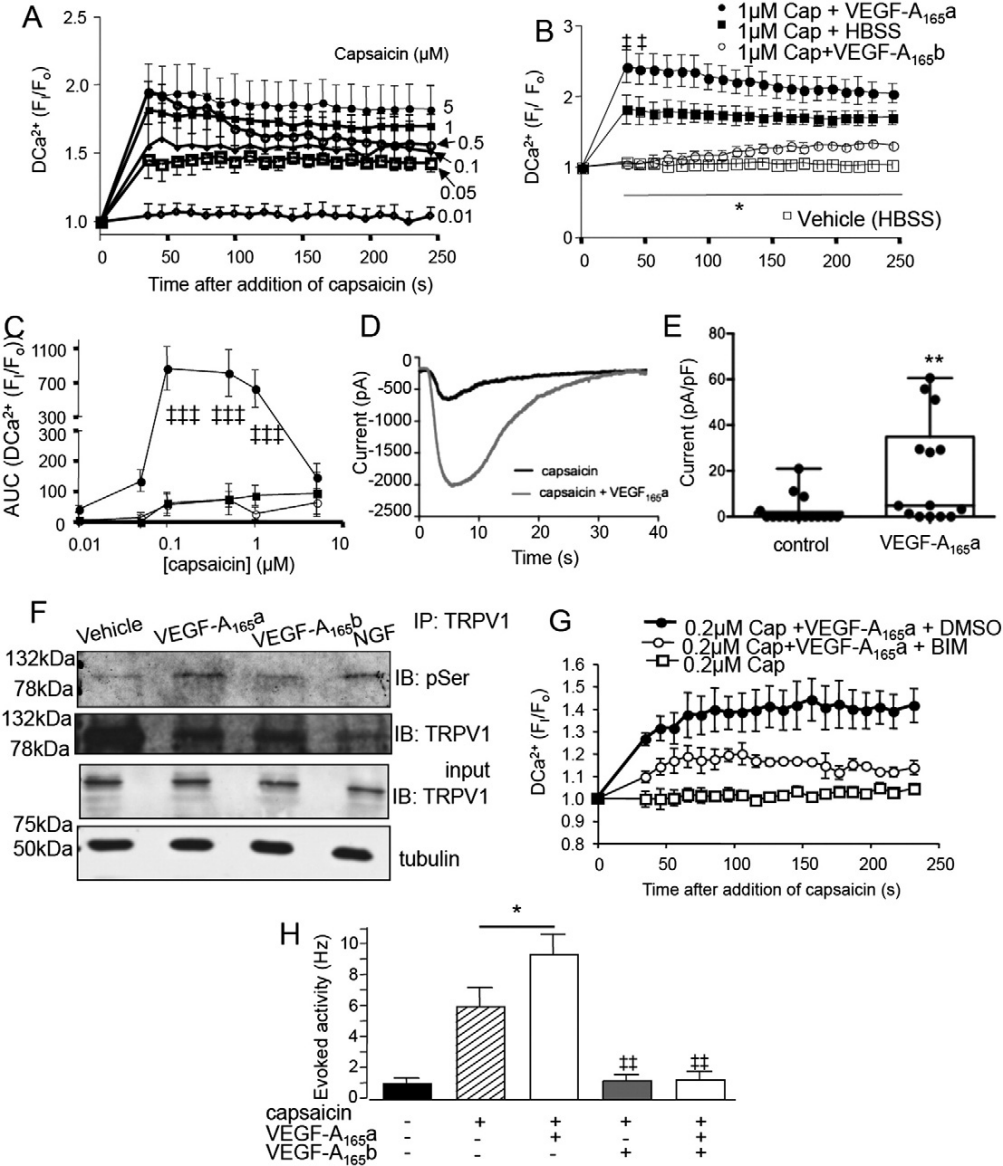
VEGF-A modulated TRPV1-agonist evoked responses in dorsal root ganglion neurons. A. Capsaicin stimulated a concentration-dependent increase in intracellular calcium in DRG neurons. B. This was increased by rhVEGF-A_165_a, and reduced by rhVEGF-A_165_b (mean ± SEM, n = 3–7). C. Treatment of rat DRG neurons with rhVEGF-A_165_a increased capsaicin-stimulated calcium influx (area under the curve of the calcium responses shown in Fig. 8B) compared with capsaicin alone or rhVEGF-A_165_b (2 way ANOVA main effect of drug p = 0.0051). The bell shaped concentration–response curve displays TRPV1 desensitization at higher capsaicin concentrations (5 μM). D. Example of a digitized trace of raw capsaicin-evoked current in the presence (gray) and absence of capsaicin. E. Capsaicin-evoked currents in primary DRG neurons were significantly larger in neurons incubated in VEGF-A_165_a overnight compared to vehicle treated neurons (box and whisker plots showing median, range, min and max). F. rhVEGF-A_165_b treatment enhanced TRPV1 serine phosphorylation in 50B11 immortalized DRG cells. IP of protein with TRPV1 antibody followed by IB with anti-pSer antibody showed rhVEGF-A_165_a, but not rhVEGF-A_165_b-mediated phosphorylation of TRPV1. (NGF treatment = positive control). G. Whereas 0.2 μM capsaicin alone did not alter intracellular calcium itself, overnight treatment with rhVEGF-A_165_a + 0.2 μM capsaicin resulted in a robust sustained increase in response to capsaicin, which was blocked by treatment with the PKC inhibitor BIM1 (2 way ANOVA main effect of drug p = 0.0003). H. Low concentration capsaicin (concentration at terminals ~ 10 nM) led to evoked activity from C fiber nociceptors in vivo. Capsaicin-evoked activity was increased by rhVEGF-A_165_a and blocked by rhVEGF-A_165_b. ‡, ‡‡, ‡‡‡, p < 0.05, 0.01, 0.001 respectively compared to baseline. *, **, *** = p < 0.05, 0.01, 0.001 respectively compared to other groups.

We then determined whether VEGF-A isoforms affected TRPV1 function in sensory neurons. Capsaicin induced a dose dependent increase in intracellular calcium in primary DRG cells (Fig. 8A). Treatment with rhVEGF-A_165_a enhanced TRPV1-ligand (capsaicin) stimulated calcium influx (Figs. 8B & C), confirmed by patch clamping, where rhVEGF-A_165_a enhanced TRPV1-ligand induced currents (Figs. 8D & E) consistent with altered pain behavior. Capsaicin induced currents were found more frequently in primary DRG neurons incubated with VEGF-A_165_a (10/14 responders) than control (4/16, p = 0.03). rhVEGF-A_165_a, but not rhVEGF-A_165_b, caused significant TRPV1 phosphorylation in DRG cells, with no increase in overall TRPV1 expression level (Fig. 8F). Sensitization of TRPV1 is fundamental to the development of hyperalgesia (Ferrari et al., 2010) and dependent on PKC phosphorylation (Ristoiu et al., 2011). The VEGF-A_165_a-enhanced calcium response was inhibited by incubation with the PKC inhibitor bisindolylmaleiamide-1 (BIM, Fig. 8G). In vivo, low dose capsaicin evoked neuronal activity in primary afferent nociceptors, which was increased by rhVEGF-A_165_a (Fig. 8H) and was blocked by rhVEGF-A_165_b (Fig. 8H). These behavioral, cellular and in vivo physiological experiments indicate that VEGF-A_165_a-enhanced pain is at least partly mediated by enhanced sensory neuronal properties, through mechanisms that involve activation of PKC, and TRPV1 phosphorylation.

## Discussion

Clinical and experimental reports of the detrimental effects of anti-VEGF agents on neuronal integrity and pain have raised concerns over the use of such therapies as their use can result in neuronal damage, often leading to pain (Verheyen et al., 2012). VEGF-A_165_a is reported to have both pro- (Benton and Whittemore, 2003, Herrera et al., 2009, Liu et al., 2012, Malykhina et al., 2012) and anti-nociceptive effects (Grosios et al., 2004, Lin et al., 2010, Verheyen et al., 2013). We hypothesized that this conflict in the literature regarding findings on pain may be resolved by a more detailed understanding of the contributions of the alternatively spliced VEGF-A isoforms to nociception. We show herein that a controlled change in the repertoire of VEGF-A alternative splice variants in the environment around peripheral sensory neuronal fibers/terminals, using either exogenous protein or control of endogenous splicing in favor of VEGF-A_xxx_a, results in enhanced pain, and that VEGF-A_165_b can alleviate pain in neuropathy.

**Fig. 9:**
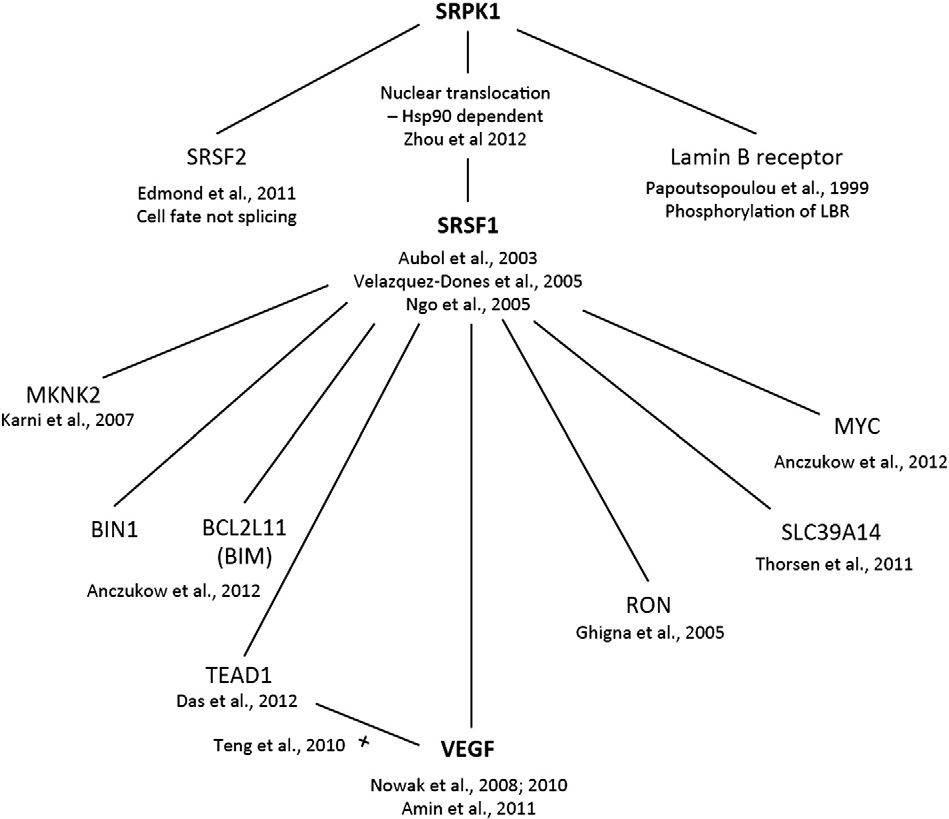
Downstream targets of the serine–arginine protein kinase SRPK1. The serine–arginine protein kinase is known to have three major downstream targets, the RNA splicing factors SRSF1 (Edmond et al., 2011), SRSF2 (Aubol and Adams, 2011, Ngo et al., 2005, Velazquez-Dones et al., 2005), and the lamin B receptor (Papoutsopoulou et al., 1999). SRPK1 activity results in Hsp90-dependent nuclear translocation of SRSF1 (Zhou et al., 2012). SRSF1 has been reported to control alternative RNA splicing of the proto-oncogene *myc*, BIM (BCL2L11) (Anczukow et al., 2012), the cation cotransporter SLC39A14 (Thorsen et al., 2011), the tumor suppressors MKNK2 and BIN1 (Das et al., 2012, Karni et al., 2007), the angiogenesis related genes RON (Ghigna et al., 2005) and TEAD1 (Das et al., 2012), and VEGF-A (Amin et al., 2011, Nowak et al., 2010, Nowak et al., 2008). TEAD1 activates VEGF-A expression (Teng et al., 2010). None of the downstream targets of SRPK1 has been implicated in nociception other than VEGF-A.

Although differential expression of several alternatively spliced growth factors has been reported after peripheral nerve injury (Amiri et al., 2009, Chen et al., 2008, Kerber et al., 2003, Kerr et al., 2010), and injured peripheral neurons show altered RNA splicing (Kiryu-Seo et al., 1998), control of pain through targeting of alternative RNA splicing has not been previously reported. We have shown, for the first time, that peripheral axotomy activates changes in alternative RNA splicing in the area of damage, where mediators in the local environment can profoundly affect neuronal properties (Djouhri et al., 2012, Obata et al., 2004), possibly through TRPV1 activation on sensory nerve fibers (Hoffmann et al., 2009), as well as in the damaged neurons themselves. Use of a specific SRPK1 inhibitor has, also for the first time, allowed RNA splicing mechanisms to be considered as a potential analgesic strategy and enabled us to identify a relationship between changes in alternative RNA splicing and pain. The serine–arginine-rich protein kinases (SRPKs) are a small kinase family with principal actions on mRNA splicing and maturation (Giannakouros et al., 2011). Of the mammalian target RNAs affected by SRPK1/2 and SRSF1-controlled splicing (Fig. 9), none have been previously implicated in pain or nociception, other than VEGF-A.

As pre-mRNA splicing inhibition affected the balance of endogenous VEGF-A isoforms and nociception, and exogenous VEGF-A isoforms modulated behaviors and neuronal properties in a similar fashion, we hypothesize that it is the balance of VEGF-A_xxx_a and VEGF-A_xxx_b that determines the net effect on nociception. A slight disruption in this balance can have profound effects on VEGFR2 function (Table 1) as both receptor number and intracellular signaling mechanisms are altered. VEGF-A_165_a and VEGF-A_165_b have the same binding affinities to VEGFR2. However, when the two isoforms are equimolar or VEGF-A_165_b is in excess (as it often is in normal tissues, data herein, (Harper and Bates, 2008, Pritchard-Jones et al., 2007)), VEGF-A_165_b can reduce VEGF-A_165_a actions by ~ 95% (Hua et al., 2010). This is brought about by competitive antagonism at VEGFR2 (Kawamura et al., 2008, Woolard et al., 2004), and reduction in receptor number (Ballmer-Hofer et al., 2011). This complex mechanism can explain why local alteration of alternative RNA splicing, with a > 60% reduction in VEGF-A_165_a mRNA in skin, induced hypoalgesia in normal animals whereas systemic low concentration VEGF-A_165_b had little effect. Conversely, increasing VEGF-A_165_a using systemic exogenous recombinant protein had clear pro-nociceptive effects on both behavior and neurons.

Increasing local VEGF-A_165_a had a robust action on a sub-population of small unmyelinated somatic nociceptors that express functional VEGF receptors and TRPV1 receptors, increasing spontaneous firing (Djouhri et al., 2006, Hulse et al., 2010b) and mechanically-evoked activity, and lowering activation thresholds, all changes indicative of peripheral sensitization of sensory neurons. All of these changes, particularly increased spontaneous firing, increase afferent barrage and induce central sensitization in the spinal cord and higher centers, leading to altered pain behaviors (hyperalgesia and allodynia) (Grubb, 1998). Peripheral administration of VEGF-A_165_a had rapid (within 5 min) effects on primary afferents in vivo, suggestive of direct VEGF-A effects on neurons. This is supported by our data that show increased Y1175 phosphorylation of neuronal VEGFR2 after nerve injury, and by the direct modulation of TRPV1 currents in isolated neurons. Neuronal properties in intact afferent fibers can be affected by growth factor/inflammatory mediator actions at both receptor terminals, as a result of neuroinflammation caused by degeneration of adjacent fibers (Djouhri et al., 2012, Obata et al., 2004), and by mechanical stimulus-enhancement of endothelin hyperalgesia, mediated through endothelial cell ATP release and nociceptor sensitization (Joseph et al., 2013, Joseph, 2014). VEGF effects on neurons are unlikely to be entirely mediated through indirect vascular effects, as local blood flow was unaffected by the VEGFR antagonists that reduced nociceptive thresholds. We cannot completely exclude a contribution from the vasculature in the mechanical behavioral effects of VEGF-A_165_a (Chen et al., 2014, Joseph et al., 2013). This mechanism could contribute only in part to the pro-nociceptive effects that we report, as the actions of VEGF-A_165_a on cultured neurons, and in vivo demonstrate that VEGF-A_165_a exerts direct sensitizing effects on neurons that are independent of any mechanical stimulation, or other cells.

PLC/PKC signaling is key in peripheral nociceptor sensitization (Ferrari et al., 2010, Joseph et al., 2007), as changes in PKC activation modulate both voltage gated sodium channels (Malykhina et al., 2012, Stamboulian et al., 2010) and other key channels such as TRPV1 (Moriyama et al., 2005, Ristoiu et al., 2011, Rosenbaum and Simon, 2007). Our results show that, at least in vitro, PKC contributes to the VEGF-A_165_a modulation of TRPV1 sensitivity, possibly thereby contributing to alteration of neuronal properties/excitability. VEGF-A proteins also interact with neuropeptides in other tissues, such as somatostatin and angiotensin in the retina (Mei et al., 2012, Wilkinson-Berka, 2004) and kappa opioids in tumor angiogenesis (Yamamizu et al., 2013, Yamamizu et al., 2011) often through common downstream signaling pathways (Pan et al., 2008). Interestingly, all these neuropeptides are also implicated in nociception (Pan et al., 2008, Rice et al., 2014), suggesting that VEGF-A nociceptive signaling may also involve complex interactions with other pro-nociceptive molecules, in addition to its direct effects.

VEGF-A_165_b has actions on nociception that involve TRPV1, a key molecule in the sensitization of neurons leading to chronic pain states (Levine and Alessandri-Haber, 2007). VEGF-A_165_a exerts direct effects on agonist-induced TRPV1 channel opening (Figs. 8D, E), TRPV1-evoked calcium signaling and TRPV1 phosphorylation in isolated DRG neurons (Fig. 8), and alters neuronal properties in neurons co-expressing functional TRPV1 receptors resulting in peripheral mechanical sensitization (Fig. 3) suggesting direct modulation of neuronal TRPV1. It is therefore somewhat surprising that VEGF-A_165_a altered mechanical but not thermal thresholds in the normal animal, given that TRPV1 is well-known as a thermal transducer molecule (Caterina et al., 1997). Local capsaicin can however cause peripheral mechanical sensitization of cutaneous (Li et al., 2008, Ren et al., 2005, Ren et al., 2006, Wang et al., 2011), deep tissue and visceral afferents (Kiyatkin et al., 2013, Lam et al., 2009). The mechanism(s) through which TRPV1-dependent peripheral mechanical sensitization of afferents occurs are not known, but may be a consequence of altered nociceptor excitability, rather than directly affecting mechanotransduction per se (Malykhina et al., 2012, Raouf et al., 2012). Heterodimerization of TRPV1 with TRPA1 (Akopian, 2011), a molecule implicated in mechanical sensitization of primary afferents (Dunham et al., 2008, Lennertz et al., 2012), may explain, in part, the TRPV1 agonist effects on mechanical nociception. Of course, we cannot exclude the possibility of a contribution of an indirect effect through TRPV1 expressed elsewhere, particularly as TRPV1 is expressed in vascular and connective tissues (Fernandes et al., 2012), but the weight of evidence suggests a direct effect is at the very least a major contributor. In addition to a peripheral sensitizing action, VEGF-A_165_a could exert central effects, as both TRPV1 knockout and antagonist interventions (Fernandes et al., 2011) can also affect central TRPV1 receptor function. Indeed our results suggest that this is the case under sensitized but not normal conditions as contralateral effects of VEGF-A_xxx_a and b were seen in nerve injured animals but not in normals, (Fig. 5) (Hulse et al., 2012).

Pain is an expected consequence of neuronal damage, as the resulting local neuro-inflammatory responses alter the properties of peripheral sensory neurons. Neuroprotective therapeutic strategies are therefore thought to be good for both analgesia, and functional loss associated with neuronal damage. VEGF is known to be neuroprotective. It has thus been suggested that anti-VEGF therapies cause pain through blockade of the neuroprotective actions of VEGF (Verheyen et al., 2013, Verheyen et al., 2012). However, while both VEGF-A_165_a (Rosenstein and Krum, 2004, Storkebaum et al., 2004) and VEGF-A_165_b are neuroprotective for peripheral and central neurons (Beazley-Long et al., 2013) our findings show that only VEGF-A_165_b is anti-nociceptive. Thus the pain associated with anti-VEGF and anti-VEGFR therapies is unlikely to be *entirely* attributable to a loss of neuroprotective effect, but probably also involves modulation of nociception by VEGF-A isoforms. A more likely explanation for the difference in the effects of VEGF-A isoforms on pain behavior, and sensory neuronal function is a multifactorial process including alteration of the balance of isoforms present, different downstream actions on VEGFR2, and/or effects on central processing of nociceptive inputs, as well as neuroprotection.

These findings have important implications for the treatment of conditions in which VEGF-A drives pathology. VEGFR2 upregulation both ipsi- and contralateral to nerve injury in nociceptive neurons, involved in the establishment of chronic pain (Ferrari et al., 2010) may imply that VEGF-A is an important molecule in the protective priming of nociceptive systems around the body that can occur as a result of peripheral nerve damage or inflammation (Donaldson, 1999, Koltzenburg et al., 1999b). Consideration will need to be given as to whether isoform-specific VEGF-A supplementation might itself be used as an analgesic therapy. Early intervention to prevent changes in VEGF-A mRNA alternative splicing in pathological conditions may contribute to the *prevention* of the development of pain, in addition to being valuable in the treatment of existing pain. These findings open up the possibility of developing a novel class of analgesic agents based on controlling the splice regulatory mechanisms determining the balance of VEGF-A isoforms.

## Author contribution statement

NB-L, RPH, HK, JP, DOB, HB, MVG, YQ, & ESF performed research. KBH, YQ, SJH, ADdG, & SDB provided research materials (antibodies, tissues, animals). NB-L, RPH, JH, SJH, AJC, SDB, DOB, & LFD designed the research, and analyzed data. DOB and LFD wrote the manuscript with contributions and final approval from all other authors. The data reported in this manuscript are available from the corresponding authors.
